# Phytochemical and Psychotropic Research of Motherwort (*Leonurus cardiaca* L.) Modified Dry Extracts

**DOI:** 10.3390/plants10020230

**Published:** 2021-01-25

**Authors:** Oleh Koshovyi, Ain Raal, Igor Kireyev, Nadiya Tryshchuk, Tetiana Ilina, Yevhen Romanenko, Sergiy M. Kovalenko, Natalya Bunyatyan

**Affiliations:** 1 Department of Pharmacognosy, National University of Pharmacy, 53-Pushkinska str., 61002 Kharkiv, Ukraine; oleh.koshovyi@gmail.com (O.K.); ilyinatany86@gmail.com (T.I.); gnosy@nuph.edu.ua (Y.R.); 2 Institute of Pharmacy, Faculty of Medicine, University of Tartu, Nooruse 1, 50411 Tartu, Estonia; 3 Department of Pharmacology and Pharmacotherapy, National University of Pharmacy, 53-Pushkinska str., 61002 Kharkiv, Ukraine; ivkireev1026@gmail.com (I.K.); trychshuk1@gmail.com (N.T.); 4 Department of Organic Chemistry, V.N. Karazin Kharkiv National University, 4 Svobody Sq., 61022 Kharkiv, Ukraine; kovalenko.sergiy.m@gmail.com; 5 Department of Pharmaceutical Technology and Pharmacology, Federal State Autonomous Educational Institution of Higher Education I.M. Sechenov First Moscow State Medical University of the Ministry of Health of the Russian Federation (Sechenov University), 8-2 Trubetskaya str., 119991 Moscow, Russia; ndbun@mail.ru; 6 Clinical Pharmacology Center, Federal State Budgetary Institution ‘Scientific Centre for Expert Evaluation of Medicinal Products’ of the Ministry of Health of the Russian Federation, Petrovsky Boulevard 8, bld. 2, 127051 Moscow, Russia

**Keywords:** *Leonurus cardiaca* L., tincture, dry extract, modification, amino acid, biological active substances, psychotropic activity

## Abstract

The prospect of creating a new medicine with psychotropic activity is shown as a result of studying the chemical composition and pharmacological activity of modified dry extracts of motherwort (*Leonurus cardiaca* L.) tincture. The most promising substances were the dry extracts, modified by adding small amounts of arginine, valine, phenylalanine, glycine, lysine, and alanine. A total of 15 main phenolic substances were found in the extracts, and eight of them were identified. There were also 10 hydroxycinnamic acids in these extracts, three of which were identified (chlorogenic, caffeic, and rosmarinic acids). The dominant hydroxycinnamic acids were chlorogenic and caffeic acids. Among flavonoids, catechin, hyperoside, and rutin were identified. It should be noted that the extracts had a significant content of ellagic acid. On the basis of the results of the phytochemical analysis of the extracts, it can be concluded that the composition of phenolic compounds does not differ significantly, and the main differences are related to amino acids, which obviously have an impact on the overall pharmacological effect. The results obtained indicate the presence of anxiolytic activity in the motherwort extracts studied in complex with amino acids. The extracts with glycine, valine, and arginine were more effective in reducing anxiety in animals.

## 1. Introduction

In the conditions of the modern rhythm of life, disorders of the central nervous system (CNS) associated with excessive excitability and increased anxiety occur more commonly. Chronic stress and emotional overload lead to the appearance of symptoms of neurogenic and psychosomatic diseases. According to World Health Organization (WHO) data (2017) [1], depression ranks first in the world among other diseases, affecting about 300 million people, and bipolar disorder takes second place. According to the results of modern research, 60–90% of visits to doctors are associated with stressful conditions. For the medical correction of such disorders, sedatives and anxiolytics are mainly used. When choosing sedative medicines, more than 80% of the population prefers herbal products [1,2,3].

Motherwort herb (*Leonurus cardiaca* L.) is one of the most widely used sedative medicinal plants [4]. The literature provides significant scientific data on the study of the chemical composition of representatives of the *Leonurus* genus. In general, the structures of about 270 substances have been isolated and determined from this genus. They are represented by alkaloids [5,6] monoterpenoids, sesquiterpenoids, diterpenoids, triterpenoids, iridoids [7], flavonoids [8], phenylpropanoids, steroids [9], and cyclic peptides. Most scientific primary sources focus on the study of alkaloids (leonurine and stachydrine) and diterpenoids (147 labdanum diterpenoids). The aerial parts of representatives of the *Leonurus* genus have been studied to a greater extent [8,10,11,12].

Motherwort preparations were first used in medical practice as cardiotonic agents regulating the heart activity rhythm, in angina pectoris, cardiovascular neuroses, and the initial stages of hypertension [13]. It was only at the beginning of the 20th century that it started to be used as a sedative drug, increasing the processes of inhibition or decreasing the processes of excitation in the CNS. Currently, it is used in cardiosclerosis, increased blood pressure, angina pectoris, myocarditis, cardioneurosis, excessive nervous excitability, especially when it is associated with clymacteria in women and with prostatic hypertrophy in men, neurasthenia accompanied by insomnia, a sense of tension, and increased reactivity, as well as vegetative neurosis [14,15]. Motherwort preparations are indicated in epilepsy, hypomenstrual syndrome, Basedow’s disease, gastrointestinal diseases, etc. [8,16,17,18,19]. Modification of herbal medicines leads to an increase in their activity and appearance of new pharmacological effects of biologically active substances (BAS), for example, anxiolytic and diuretic activity.

Herbal agents or dry raw materials constitute many medicines. One of the most common medicinal products based on this medicinal plant is the motherwort tincture [16]. The method of obtaining the motherwort tincture is well known [16,20]. However, the disadvantage of this drug is the instability of the chemical composition and, as a result, the pharmacodynamics. In addition, in the manufacture of tinctures, ethyl alcohol is used as an extractant, which not only limits the number of patients who can use this drug (children, pregnant women, nursing mothers, persons whose activities require increased attention, etc.), but, in turn, is also able to affect the CNS and modulate the action of the drug itself. Therefore, these categories of patients are recommended to take water extracts of this raw material, but these dosage forms are not standardized, are poorly stored, and have a different chemical composition. In this regard, the development of standardized medicines based on tincture of the motherwort herb, which do not contain ethanol in its composition, is an urgent task [21,22].

The technology for obtaining a dry tincture of motherwort herb was previously substantiated. It involves replacing alcohol with a dry excipient (sucrose, mannitol, sorbitol, etc.). The resulting product has favorable technological parameters: satisfactory bulk properties, less hygroscopicity, and stability in storage [21,22].

Amino acids in extracts are able to form salts, complexes, and conjugants with other BAS groups, for example, with phenol compounds, and this changes their physicochemical properties, affects their solubility, bioavailability, and total pharmacological effect, and leads to an increase in their activity and the appearance of new pharmacological effects [23,24,25,26]. Therefore, modification of the motherwort tincture using amino acids and addition of excipients to create new dry extracts is an urgent task of modern pharmacy.

The aim of the work was to study the phytochemical profile and the psychotropic activity of modified dry extracts of motherwort tincture.

## 2. Results

### 2.1. Phytochemical Research

The motherwort tincture, which was used to obtain modified extracts, fully met the requirements of SPhU 2.0 “Motherwort tincture N”. The dry residue in the tincture was 2.04% ± 0.03%, while the content of phenolic compounds in the dry residue of the tincture was 8.17 ± 0.06%.

The modified dry extracts obtained were greenish-brown powders with a characteristic odor. As a result of thin-layer chromatography (TLC) analysis of the dry extracts obtained, the following groups of biologically active substances (BAS) were identified: flavonoids (rutin and hyperoside), derivatives of hydroxycinnamic acids (chlorogenic, caffeic, and rosmarinic acids), and iridoid compounds (harpagide, harpagide acetate, and ayugoside).

The extracts obtained were analyzed by HPLC (Table 1). As a result of the study, motherwort extracts showed a rather similar chemical composition of phenolic compounds, since the same tincture was used as the primary raw material. On the other hand, in many cases, the difference between the contents of BAS in extracts with and without amino acids was considered to be extremely statistically significant (*p* < 0.0001). In the extracts, 15 main substances of phenolic nature were found, and eight of them were identified. There were also 10 hydroxycinnamic acids in these extracts, three of which were identified (chlorogenic, caffeic, and rosmarinic acids). The dominant hydroxycinnamic acids were chlorogenic and caffeic acids. Among flavonoids, catechin, hyperoside, and rutin were identified. It should be noted that the extracts had a significant content of ellagic acid.

The chromatograms of the modified extracts were rather similar. If stable covalent connections were formed, they were seen as new substances on the chromatograms. According to our hypothesis, this allows suggesting that amino acids formed conjugates, salts, or complexes with phenolic compounds and iridoids, which were easily destroyed by the acid of the chromatographic system.

In addition, the quantitative content of flavonoids, iridoids, and polyphenols was determined in the extracts using a spectrophotometric method (Table 2 and Table 3). The obtained results of phytochemical analysis were used for standardization of the obtained extracts, for which the study of psychotropic activity was carried out. The content of the main groups of BAS in the extracts obtained did not differ statistically significantly (*p* = 0.27–0.64), since the raw material—the motherwort tincture—was the same and was taken in the same quantities. They were compared with the literature data from phytochemical studies of motherwort tincture by other authors [11,17]. According to the results of the phytochemical analysis of extracts, it can be concluded that the composition of phenolic compounds did not differ significantly, and the main differences were related to amino acids, which obviously had an impact on the overall pharmacological effect.

### 2.2. Research on Psychotropic Activity of the Extracts

**Figure 1:**
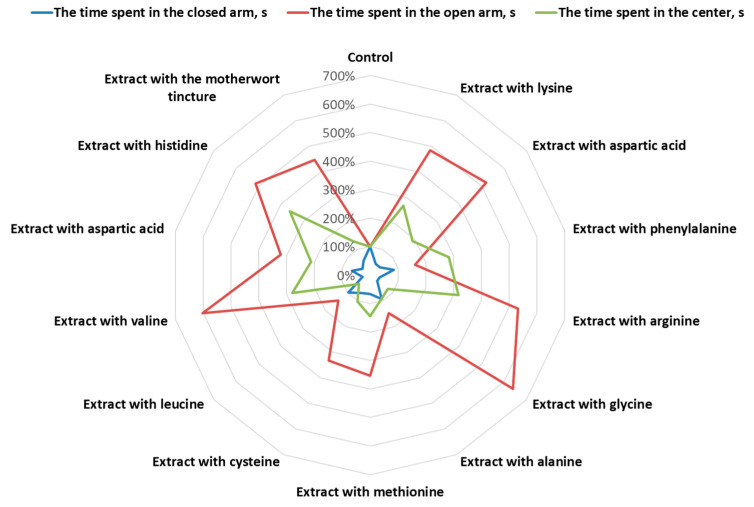
The results of distribution of activity types of the motherwort extracts on behavioral responses of laboratory rats in the open-field test.

The results of the studies of the effect of the motherwort extracts modified with amino acids on the behavioral responses of laboratory rats in open-field tests are given in Table 4 and Figure 1.

The doses of amino acids in the obtained extracts were significantly lower than those described in the literature, and they provided a certain pharmacological effect. In this regard, to study them as individual substances was impractical.

**Figure 2:**
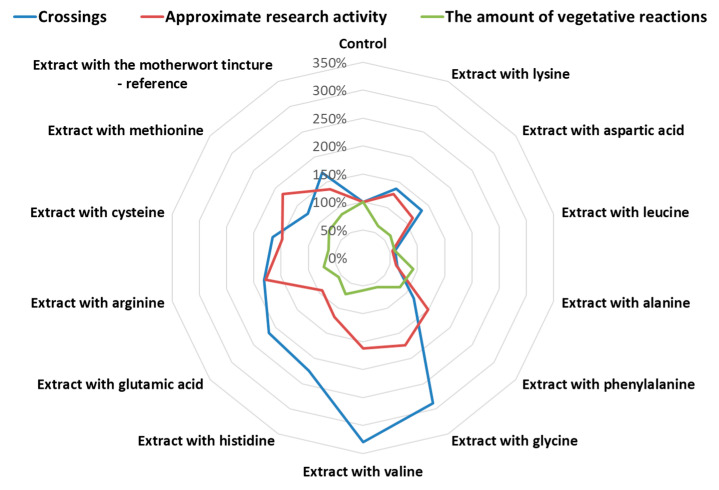
The results of distribution of activity types of the motherwort extracts in the elevated plus maze test.

The results of the studies of the modified dry extracts of motherwort in the elevated plus maze tests are given in Table 5 and Figure 2.

The manifestation of motor activity in the open-field test is determined by the number of squares crossed by the experimental animal. According to the results obtained, the administration of the motherwort extracts in the complexes with valine, glycine, histidine, and glutamic acid resulted in a significant increase in this indicator by 229%, 188%, 124%, and 115%, respectively. A significant increase in the number of squares crossed compared to control animals (*p* < 0.01) was observed in groups of rats taking the extracts of motherwort in combination with arginine (by 81%) and cysteine (by 66%), as well as the tincture of motherwort (by 69%). At the same time, in groups of animals receiving the dose of the motherwort extract with leucine and alanine, this activity was decreased by 41% and 37% compared to the control group. The remaining motherwort extracts studied in the combination with amino acids showed a slight tendency to increase the index of the motor activity.

An increase in the number of upright postures was observed in the groups of animals receiving motherwort extracts in the complexes with lysine (by 41%), aspartic acid (by 41%), glycine (by 68%), valine (by 54%), arginine (by 48%), methionine (by 52%), and cysteine (by 41%). These changes were significant at *p* < 0.05 and *p* < 0.01 compared to the control. The introduction of the motherwort extracts in combination with phenylalanine and histidine, as well as the tincture of motherwort, led to an increase in the index by 38% (*p* > 0.05), 36% (*p* < 0.05), and 37% (*p* < 0.05), respectively. The motherwort extract in combination with glutamic acid showed an insignificant increase in the number of postures, but the result was not reliable at the significance level of *p* < 0.05. In the groups of animals that received the motherwort extract in combination with leucine and alanine, this indicator was 47% and 26% lower, respectively, compared to the control.

The exploratory activity shown in the number of holes examined in the field significantly increased in animals taking the motherwort extracts in the complexes with phenylalanine, glycine, valine, arginine, cysteine, and methionine by 65%, 82%, 76%, 133%, 60%, and 67%, respectively, compared to the control group, and decreased in animals receiving the motherwort extract in combination with leucine, alanine, and glutamic acid by 43%, 57%, and 47%, respectively. The introduction of the motherwort extracts in the complexes with aspartic acid and histidine led to an unreliable decrease in the number of the holes examined, while the tincture of motherwort increased the index by 33% (*p* > 0.05).

The motherwort extracts in combination with arginine, glycine, valine, methionine, phenylalanine, and cysteine significantly increased the number of exploratory responses compared to the control. At the same time, the extracts of motherwort in combination with glycine and arginine had a similar effect on this indicator compared to the group of animals receiving the motherwort tincture, thus increasing the number of responses (at *p* < 0.05).

A statistically significant decrease in the animal’s emotional response, such as the number of defecation events, was observed in groups receiving the motherwort extract in combination with lysine (by 53%), aspartic acid (by 53%), leucine (by 53%), glycine (by 47%), glutamic acid (by 57%), and cysteine (by 41%). The remaining complexes studied did not show a significant effect on the animal’s response.

Assessing the effect of the complexes under study on the number of urinations revealed a statistically significant decrease in the indicator in groups of animals receiving the motherwort extracts in combination with leucine (by 42%), alanine (by 42%), glycine (by 50%), valine (by 58%), glutamic acid (by 50%), and arginine (by 46%).

Significant changes in the number of grooming acts, i.e., a decrease, were observed with the introduction of the motherwort extracts in combination with alanine and methionine by 55% and 46%, respectively, compared to the control group (*p* < 0.05).

Assessing the total vegetative manifestation of animal responses under stressful conditions revealed a significant decrease in indicators (*p* < 0.05) in groups of animals receiving the motherwort extracts with lysine (by 37%), aspartic acid (by 37%), and cysteine (by 37%) due to a reduction in the number of acts of defecation, the extracts with valine (by 42%) and arginine (by 38%) due to a reduction in the number of urinations, the extracts with leucine (by 42%), glycine (by 42%), and glutamic acid (by 44%) by reducing the number of defecations and urinations, and the extract with histidine (by 38%).

The ability of drugs to increase the motor activity of animals may indicate their stimulating effect on the CNS. This effect is typical for medicines with nootropic, adaptogenic, normotimic, actoprotective, and other activities. At the same time, there were no statistically significant differences in other types of behavioral reactions against the background of these pharmacological agents.

Some compounds insignificantly increased the activity of rat defecation in the open-field test. This may indicate activation of the parasympathetic peripheral nervous system—a common reaction of higher animals to stress. However, compounds containing leucine, glycine, and valine had a vegetative-stabilizing effect; therefore, it could be argued that these compounds had a stress-protective effect.

## 3. Discussion

Generally, the phytochemical profile of motherwort has been studied very well and described in several reviews [8,11,19]. Different alkaloids, diterpenoids, triterpenoids, iridoid glycosides, flavonoids, sterols, essential oils, phenylpropanoids, phenolic acids, glycosides, tannins, peptides, etc. have been identified in the aerial parts of motherwort by various authors, as described in [8,11]. Thus, it would be rather complicated to find something new in the chemical composition of the species. However, methoxy apigenin, ellagic acid, and catechin found in our study were not previously mentioned in motherwort. The sedative activity of motherwort is theoretically derived from the iridoids, whereas the alkaloid leonurine does not play an important role in central nervous system activity but could be a potential compound for vomiting and nausea [8]. Furthermore, the hypotensive and calming effect of motherwort may be caused by iridoids [11]. The relationship between the chemical composition of motherwort and its pharmacological effects is not yet clear.

In the extracts, 15 main substances of phenolic nature were found, and eight of them were identified. There were also 10 hydroxycinnamic acids in these extracts, three of which were identified (chlorogenic, caffeic, and rosmarinic acids). The dominant hydroxycinnamic acids were chlorogenic and caffeic acids. Among flavonoids, catechin, hyperoside, and rutin were identified. It should be noted that the extracts had a significant content of ellagic acid.

The absorption maxima of the dry modified extracts of dog nettle were firstly in the range of 281–292 nm and secondly in the range of 324–331 nm (Table 3). The largest visually noticeable changes in the ultraviolet (UV) spectra of the extracts were noticeable in the range of 281–292 nm, which indicates the interaction of amino acids with an aromatic group. In addition, when amino acids were added, hypo- and hyperchromic shifts occurred in absorption maxima. The ratio of absorption in these peaks varied depending on the amino acid, which indicated the formation of different complexes.

The tincture or oil extract of motherwort was confirmed in clinical trials to reduce anxiety and stabilize emotional state due to the content of iridoids, as in other antianxiety medicinal plants [8].

The motherwort extracts with alanine and leucine had a statistically significant inhibitory effect on the motor and exploratory activity of animals. They reduced the number of squares crossed by 37% and 41% (*p* < 0.01), while the sum of upright postures and the holes examined decreased by 57% and 43% (*p* < 0.01).

Such an effect on the behavior of rats can be regarded as anxiolytic. This effect is typical for a number of neurotropic drugs: anxiolytics, tranquilizers, stress protectors, antidepressants, and medicines with normotimic, neuroleptic, and other activities. This effect of the pharmacological agent is predictable due to the presence of a pronounced anxiolytic action in its composition. Iridoids have previously been shown to have anxiolytic activity [27]. Alanine is a CNS factor that prevents excessive excitation of the nervous tissue and causes inhibition of excitation. This amino acid shows its anxiolytic effect through interaction with sites of the glutamate N-methyl-D-aspartate (NMDA) receptor, the glycine co-agonist site on the NMDA receptor, and receptors of γ-aminobutyric acid of type A and C, as well as by inhibiting the reverse neuronal capture of gamma-aminobutyric acid (GABA) and enhancing GABAergic neurotransmission [28]. It is probable that alanine potentiates the anxiolytic effect of the motherwort extract.

The results of this experiment confirm the potential benefit of using the motherwort extracts with alanine in the treatment of psychovegetative disorders as a sympathetic tonic.

Thus, the motherwort extracts in the complexes with valine, glycine, arginine, and cysteine were the most active; they led to an increase in the motor and exploratory activity of the experimental rats, while reducing the indicators of the animals’ emotional state.

Animals of the intact control group (IC) spent most of their time in dark compartments (arms). The percentage distribution of time (dark arms vs. light arms vs. central area) for animals of the IC group was 85.4% vs. 11.0% vs. 3.6%. This result generally corresponds to the typical picture of rodent behavior in a new unfamiliar space and corresponds to the literature data [27,29].

The first group included three pharmacological agents: the motherwort extracts with phenylalanine, alanine, and leucine. Against the background of these compositions, there was no statistically significant decrease in the time spent by rodents in the dark arms, and the indicator of the time during which the animals were in the light compartments did not increase. This indicates that these agents did not eliminate the anxiety in rats, which arose in connection with their stay in a new unknown brightly lit space.

The group of extracts with a moderate antianxiety activity included the motherwort tincture itself, as well as the motherwort extracts with methionine and aspartic acid. These compositions had a mild anxiolytic effect, statistically significantly reducing the time spent by rats in the dark arms and increasing the time spent in the light ones. Therefore, the tincture reduced the first indicator by 45.14% (*p* < 0.01 against the IC indicator), and the second indicator was increased 3.5-fold (*p* < 0.01). Under these conditions, the mixtures of the motherwort extracts with methionine and aspartic acid did not have a more pronounced antianxiety effect than the tincture.

The antianxiety effect of compositions of the motherwort extracts with arginine, histidine, glycine, and valine appeared to be the most expressive in these conditions (Figure 1). Each of these agents reduced the time spent by animals in dark compartments by 64.97%, 66.01%, 67.79%, and 72.57%, respectively (*p* < 0.01 vs. IC). In addition, against the background of the action of these pharmacological agents, the time spent in the light arms increased by 4.3-, 4.1-, 5.4-, and 5.0-fold, respectively (*p* < 0.01 against IC). Thus, the anxiolytic effect of these compositions according to the time indicators in dark and light arms statistically significantly (*p* < 0.01) exceeded the similar effect of the motherwort tincture. This may indicate the potentiating effect of amino acids (glycine, valine, arginine, and histidine) included in these extracts.

According to the literature [28], the ability of pharmacological agents to reduce the time spent by rodents in dark compartments by more than 50%, as well as to increase the time of their staying in light compartments by 2–4 times, can be regarded as an effect inherent to true anxiolytics. Thus, taking into account the results obtained, the motherwort extracts with amino acids (glycine, valine, arginine, and histidine) can be considered as promising products with antianxiety activity.

The results obtained indicate the presence of anxiolytic activity in the motherwort extracts studied in the complexes with amino acids. The extracts with glycine, valine, and arginine were more effective in reducing anxiety in animals. The advantages of the extracts studied include the absence of action on the muscle tone and coordination of movements.

Among the motherwort extracts studied, those in combination with alanine and leucine exhibited a pronounced anxiolytic effect. These compounds had anxiolytic activity compared to the control; however, their antianxiety activity was comparable to the reference drug.

## 4. Materials and Methods

### 4.1. Plant Material

The study object was the officinal tincture of motherwort (batch 150416, LLC LLC DKP “Vishpha Pharmaceutical Factory”, Zhytomyr region, village Stanishovka, Zhytomyr region, Ukraine), and dry extracts were obtained from this tincture.

To standardize the motherwort tincture, we used SPhU 2.0 “Motherwort tincture N” containing indicators such as the description of properties, and the following identification parameters were normalized: the content of ethanol (64–69%), dry residue (not less than 1.5%), and flavonoids (not less than 0.01% calculated with reference to hyperoside) [20].

### 4.2. Reagents

For modification of the motherwort tincture, a number of amino acids were used: lysine, aspartic and glutamic acids, leucine, alanine, phenylalanine, glycine, valine, histidine, arginine, cysteine, and methionine (a set of amino acids No. 2 big TU 6-09-3147-91, Chemical reagent kit).

Sucrose (Compri Sugar O, Witec industrial, Odessa, Ukraine) and ethanol (Biolik, Melitopol, Ukraine) were used to obtain the extracts.

The following reagents of the class “chemically pure” and “chemically pure for analysis” were used for TLC, HPLC, and spectrophotometric studies: glacial acetic acid (100063.2500, Merck, Darmstadt, Germany), ethyl acetate (Khimreserv, Kiev, Ukraine), chloroform (102444.1000, Merck), methanol (106007.2500, Merck); dimethylaminobenzaldehyde (156477 Sigma-Aldrich, St. Louis, MO, USA), hydrochloric acid (141020.1214, Merck), hydroxylamine (255580 Sigma-Aldrich, USA), and aluminum oxide (010091, Reakhim, Kharkiv, Ukraine).

Standard substances manufactured by Sigma-Aldrich, USA (chlorogenic acid C3878, caffeic acid C0625, rutin hydrate R5143, ellagic acid E2250, quercetin 3-d-galactoside 83388, rosmarinic acid R4033, (+)-catechin hydrate C1251, and 8-acetylharpagide SMB00118) were used for analysis.

### 4.3. Equipment

The obtained extracts were obtained with the use of a dry oven GP-20 (Ukraine) and vacuum circulating apparatus (ZOIBKD, Zhengzhou, Henan, China).

Studies of phenolic compounds of the extracts were performed by HPLC on a Shimadzu LC20 Prominence liquid chromatograph in a modular system equipped with a four-channel pump LC20AD, column thermostat STO20A, and automatic sampler SIL20A.

The quantitative content of the basic groups of BAS (total polyphenols, flavonoids, and iridoids) in the extracts were determined via absorption spectrophotometry using the Evolution TM 60S UV–visible light spectrophotometer (Thermo Fisher Scientific, Waltham, MA, USA).

### 4.4. Obtaining the Extracts

To modify the motherwort tincture and obtain modified dry extracts, a number of amino acids were used: lysine, aspartic and glutamic acids, leucine, alanine, phenylalanine, glycine, valine, histidine, arginine, cysteine, and methionine (a set of amino acids No. 2 big TU 6-09-3147-91, Chemical reagent kit). The formation of complexes could be concluded from the following observations: the physical properties of the extracts changed to become better soluble in water; the TLC profile of the extracts changed, with new spots appearing that were not there before; the total UV spectrum of the extracts changed; when the extracts were analyzed using an amino acid analyzer, it could be seen that the amount of free amino acid was less than that added to the extract.

The technology for obtaining a dry tincture of motherwort herb was previously substantiated [21,22]. It involves replacing alcohol with a dry excipient (sucrose, mannitol, sorbitol, etc.). The resulting product has favorable technological parameters: satisfactory bulk properties, less hygroscopicity, and stability in storage. To 50 mL of the motherwort tincture (44.97 g), the corresponding amino acids were added in a triple equimolar amount in relation to the amount of phenolic compounds (lysine hydrochloride (0.273 g), aspartic (0.187 g) and glutamic (0.220 g) acids, leucine (0.197 g), alanine (0.133 g), phenylalanine (0.250 g), glycine (0.112 g), valine (0.175 g), histidine hydrochloride (0.287 g), arginine (0.262 g), cysteine (0.182 g), and methionine (0.223 g)). The resulting mixture was left to infuse for a day to obtain complexes of phenolic compounds with amino acids, after which the solution was evaporated to 1/20 of the previous volume at a temperature of 85–95 °C in a vacuum circulating apparatus with a vacuum of 680–700 mmHg, and 20–30 g of sucrose was added. The mixture was dried, and sucrose was added (44.97 g) before powdering in a mortar until a homogeneous mass was formed. The obtained extracts were dried in a dry oven at the temperature of 50–60 °C to a moisture content of no more than 5% [20,30,31]. A dry extract based on tincture and sucrose was used as a reference drug, which was obtained in a similar way.

### 4.5. TLC Analysis of the Extracts

The TLC method was used for preliminary analysis and identification of the main active substances in extracts. Substances having a phenolic nature were identified with reliable samples of flavonoids and hydroxycinnamic acids using the system of solvents of glacial acetic acid R–water R–ethyl acetate R (20:20:60). The chromatogram was developed using dimethylaminobenzaldehyde solution; then, the plate was heated at a temperature of 100–105 °C for 10 min until the appearance of spots and examined in daylight [20,32,33].

Iridoids were identified using the TLC method with reliable samples in the system of solvents of chloroform–methanol–water (80:2:0.1) by the ascending method. The chromatogram was developed using Stahl reagent (1.0 g of *p*-dimethylaminobenzaldehyde mixed with 50.0 mL of acetic acid, 5.0 mL of phosphoric acid, and 100.0 mL of water) [34,35].

### 4.6. Sample Preparation, HPLC-Diode-Array Detector Analysis and Quantification

A total of 50.0 mg (exact weavage) of the *Leonurus cardiaca* extracts were weighed in a 5.0 mL measuring tube and brought to the mark with 90% aqueous methanol. After 30 min in an ultrasonic bath, the sample was left at room temperature for 3–4 h. Then, the test tube was again placed in an ultrasonic bath for 15 min, before filtering the solution through a Teflon filter with a pore size of 0.45 μm in a vial for analysis.

Studies of phenolic compounds of *Leonurus cardiaca* extracts were performed by HPLC on a Shimadzu LC20 Prominence liquid chromatograph in a modular system equipped with a four-channel pump LC20AD, column thermostat STO20A, and automatic sampler SIL20A in the following conditions: size—250 mm × 4.6 mm, particle size—5 μm; column temperature—35 C; detection wavelength—330 nm (for hydroxycortic acids, glycoside flavonoids), 370 nm (for flavonoid aglycones), 280 nm (for tannins), 340 nm (coumarins); flow rate of the mobile phase—1 mL/min; volume of the injected sample—5 μL; mobile phase—eluent A, 0.1% solution of trifluoroacetic acid in water and eluent B, 0.1% solution of trifluoroacetic acid in acetonitrile. The ratio of the eluents A and B changed according to the elution time: 0–5 min—95 to 5, 5–35 min—95 → 75 to 5 → 25, 35–40 min 75 to 25, 40–60 min 75 → 50 to 25 → 50, 60–65 min—50 → 20 to 50 → 80, 65–70 min—20 to 80, and 70–85 min 95 to 5, respectively. Identification of phenolic compounds was performed as a function of the retention time of standards and UV spectral characteristics [36,37,38,39,40].

### 4.7. The Quantitative Content of the Basic Groups of BAS by Absorption Spectrophotometry

The quantitative content of the basic groups of BAS (total polyphenols, flavonoids, and iridoids) in the *Leonurus cardiaca* extracts were determined by absorption spectrophotometry on an Evolution TM 60S UV–visible light spectrophotometer (Thermo Fisher Scientific, USA) [20,36,41].

### 4.8. Determination of the Quantitative Content of Total Polyphenols

The quantitative determination of the amount of polyphenols per pyrogallol was performed using the method of State Pharmacopoeia of Ukraine (SPhU) 2.0 [20,42,43].

### 4.9. Determination of the Quantitative Content of Total Flavonoids

The content of flavonoids in motherwort extracts was determined by the spectrophotometric method calculated with reference to hyperoside according to the SPhU using initial, test, and compensation solutions [20,44,45]. The optical density of the test solution was measured in 30 min in relation to the compensation solution at a wavelength of 425 nm. The content of the amount of flavonoids with reference to hyperoside (%) was calculated using the following formula:

(A × 0.625)/m,
(1)

where A is the optical density of the test solution at 425 nm, and m is the weight of a dry motherwort extract, g. A specific absorbance of hyperoside equal to 500 was used.

### 4.10. Determination of the Quantitative Content of Iridoids

The content of iridoids in motherwort extracts was determined by the spectrophotometric method calculated with reference to harpagide acetate [29,34,46,47].

Approximately 5 g of the dry extract (accurate weight) was placed in a 100 mL round-bottomed flask, diluted with 50 mL of 40% ethyl alcohol, and heated in a boiling water bath with a reflux condenser for 45 min. The extract was cooled and filtered through a cotton swab into a 100 mL volumetric flask. The solution was diluted to the volume with 40% ethyl alcohol (Solution A). Then, 10 mL of solution A was evaporated to 5 mL. The resulting solution was quantitatively transferred to a 10 mL volumetric flask and the solution was diluted to the volume with water. It was then filtered through a glass column with a diameter of 1 cm with 3 g of aluminum oxide with a II degree of activity.

Next, 5 mL of the eluate was placed in a 25 mL volumetric flask, 5 mL of an alkaline solution of hydroxylamine was added, and it was allowed to stand for 5 min. Then, 10 mL of 1 M hydrochloric acid solution was added, before diluting to the volume with a 1% solution of iron (III) chloride in 0.1 M hydrochloric acid solution and mixing.

The optical density of the resulting solution was measured on a spectrophotometer at a wavelength of 512 nm. As a reference solution, a mixture prepared in a 25 mL volumetric flask was used, whereby 10 mL of 1 M hydrochloric acid solution was added to 5 mL of hydroxylamine solution before diluting to the volume with a 1% solution of iron (III) chloride in 0.1 M hydrochloric acid solution.

The amount of iridoids in the extract with reference to harpagide acetate (X) was calculated using the following formula:

X = (A × 25 × 100 × 100 × 100)/(56 × m × 5 × (100 − w)),
(2)

where A is the optical density of the test solution, 56 is the specific absorbance of the reference standard of harpagide acetate, m is the weight of the extract, g, and w is the moisture content of the extract, %.

### 4.11. The Study of Psychotropic Activity

The effect of motherwort extracts on the behavioral responses of laboratory white rats (weighing 200–220 g) was studied in the open-field test and in the elevated plus maze test [48] at the premises of the Central Research Laboratory of the National University of Pharmacy under standard conditions on a normal diet with free access to water and food. Animals were divided into 14 groups of six rats each, with 12 experimental groups, one control group, and one reference group.

To obtain the modified extracts, amino acids were added in very small amounts with the following therapeutic doses: lysine hydrochloride—0.3 mg/kg; aspartic acid—0.21 mg/kg; glutamic acid—0.24 mg/kg; leucine—0.22 mg/kg; alanine—0.15 mg/kg; phenylalanine—0.28 mg/kg; glycine—0.12 mg/kg; valine—0.19 mg/kg; histidine hydrochloride—0.32 mg/kg; arginine—0.29 mg/kg; —0.2 mg/kg; methionine—0.25 mg/kg. In such small doses, the amino acids in the extracts do not have any pharmacological activity; thus, there is no use to check them separately, because the results of pharmacological research would be the same as in intact animals.

The extracts studied were administered orally in the form of aqueous solutions in a dose of 50 mg/kg corresponding to the therapeutic dose of the motherwort tincture 60 min before the start of the experiment. The control animals were given the appropriate amount of a saline solution [49].

The measurement of rat behavior indicators was determined in a 100 × 100 cm open-field unit divided into 25 squares and 16 openings (“holes”). In the open-field test, the following parameters were recorded: the number of rising occasions on hindpaws—the vertical component of the orientation response, the number of crossed squares—the horizontal component, the number of peeking into holes—the hole behavior reflecting the exploratory activity, and the number of acts of defecation, urination, and grooming—indicator of the animal’s emotional state. During the test, the search was initiated by going to the center of the field and increasing the number of holes studied. The test time in the open-field test was 3 min.

The method of the elevated plus maze test is based on the fear of open space and height. A rat was placed with its face to the open arm, and then the time spent in the closed and open arms, the number of visits to the closed and open arms, and the duration of staying in the center were recorded. Indicators of anxiety included the number of entries to the open arms of the maze, the duration of staying in the open arms of the maze, the ratio of the time spent in open and closed arms, the number of crossings of the central platform, and urination, defecation, and grooming activities.

Since several laboratory animals were involved in the pharmacological research, the research was carried out sequentially. Each block of studies had its own control group, for which the calculations were made.

### 4.12. Statistical Analysis

Statistical processing of the results was performed using the Student’s *t*-test. Statistical properties of random variables with *n*-dimensional normal distribution were given by their correlation matrices, which could be calculated from the original matrices. Pharmacological research material was processed using variational statistics with the calculation of the arithmetic mean and its standard error; the reliability of the compared values was estimated using the Student, Wilcoxon, and Mann–Whitney criteria with the probability level of ≤0.05 on a computer using Statistica 6.0, Word, and Excel programs [20,50,51].

## 5. Conclusions

It can be concluded that, as a result of the phytochemical and pharmacological study of the dry extracts of motherwort (*Leonurus cardiaca* L.) tincture, which were modified with 12 amino acids, the most effective substances with anxiolytic activity were extracts modified with the addition of lysine, glycine, alanine, valine, phenylalanine, and arginine. These extracts can be the basis for the creation of new potential standardized medicines with psychotropic and anxiolytic activity. The principal hydroxycinnamic acids were chlorogenic and caffeic acids, and the extracts had a significant content of ellagic acid. Among flavonoids, catechin, hyperoside, and rutin were identified. The composition of phenolic compounds did not differ significantly, and the main differences were related to amino acids, which obviously had an impact on the overall pharmacological effect.

## Figures and Tables

**Table 1:** The results of the analysis of the modified extracts of motherwort by HPLC.

Component	The Quantitative Content in the Extract, mg/100 g, $\bar{x}\pm \Delta \bar{x}$												
	Extract with the Motherwort Tincture	With Lysine	With Aspartic Acid	With Leucine	With Alanine	With Phenylalanine	With Glycine	With Valine	With Histidine	With Glutamic Acid	With Arginine	With Cysteine	With Methionine
Derivatives of caffeic acid-1	1.79 ± 0.04 ^#^	2.37 ± 0.08	1.86 ± 0.03	1.72 ± 0.04	1.89 ± 0.03	2.17 ± 0.05	1.93 ± 0.02	2.47 ± 0.05	2.52 ± 0.04	1.67 ± 0.03	1.84 ± 0.02	1.73 ± 0.03	1.61 ± 0.02
Derivatives of caffeic acid-2	2.36 ± 0.07 *	2.17 ± 0.04	2.63 ± 0.06	1.94 ± 0.02	2.52 ± 0.04	2.85 ± 0.04	2.35 ± 0.06	2.18 ± 0.07	2.69 ± 0.03	2.02 ± 0.02	2.47 ± 0.05	2.38 ± 0.05	2.13 ± 0.08
Chlorogenic acid	76.87 ± 0.57 ^#^	65.88 ± 0.87	71.3.8 ± 0.87	78.79 ± 0.93	76.38 ± 0.76	69.74 ± 0.77	75.69 ± 0.91	79.84 ± 0.84	75.27 ± 0.99	80.16 ± 0.74	76.19 ± 0.95	75.97 ± 0.69	74.84 ± 0.97
Caffeic acid	5.58 ± 0.11 *	5.08 ± 0.17	6.15 ± 0.18	6.24 ± 0.27	5.69 ± 0.13	5.18 ± 0.15	5.82 ± 0.14	5.93 ± 0.09	5.57 ± 0.09	4.98 ± 0.06	5.29 ± 0.16	5.14 ± 0.17	5.37 ± 0.41
Derivatives of caffeic acid-3	3.68 ± 0.08 ^#^	3.39 ± 0.05	3.78 ± 0.11	3.66 ± 0.09	3.32 ± 0.08	3.79 ± 0.11	3.08 ± 0.06	3.26 ± 0.12	3.68 ± 0.05	3.58 ± 0.06	2.97 ± 0.07	3.16 ± 0.14	3.05 ± 0.21
Derivatives of caffeic acid-4	14.4 ± 0.2 ^#^	11.8 ± 0.1	13.7 ± 0.2	12.9 ± 0.2	8.9 ± 0.1	13.2 ± 0.2	15.8 ± 0.2	11.9 ± 0.2	16.4 ± 0.4	11.6 ± 0.2	13.2 ± 0.4	15.7 ± 0.4	7.5 ± 0.2
Derivatives of caffeic acid-5	1.27 ± 0.02 ^#^	0.98 ± 0.01	1.04 ± 0.01	0.87 ± 0.01	nd	1.21 ± 0.02	0.95 ± 0.01	0.93 ± 0.01	1.13 ± 0.03	1.23 ± 0.03	1.17 ± 0.02	nd	0.87 ± 0.01
Rutin	1.20 ± 0.01 ^#^	1.07 ± 0.02	1.16 ± 0.01	1.27 ± 0.01	1.31 ± 0.02	0.98 ± 0.01	1.17 ± 0.01	1.26 ± 0.01	1.17 ± 0.01	1.24 ± 0.01	1.18 ± 0.02	0.95 ± 0.01	1.01 ± 0.01
Ellagic acid	41.65 ± 0.97 **	40.87 ± 0.67	37.93 ± 0.73	40.10 ± 0.57	37.69 ± 0.83	42.38 ± 0.55	41.31 ± 0.63	40.87 ± 0.67	42.16 ± 0.29	39.17 ± 0.62	40.38 ± 0.37	40.29 ± 0.91	39.79 ± 0.72
Hyperoside	2.10 ± 0.06 *	2.28 ± 0.10	1.87 ± 0.04	2.04 ± 0.06	2.18 ± 0.04	1.98 ± 0.03	2.09 ± 0.04	2.18 ± 0.05	2.39 ± 0.06	2.18 ± 0.03	2.08 ± 0.06	2.12 ± 0.03	2.07 ± 0.03
Rosmarinic acid	1.72 ± 0.03 ***	1.54 ± 0.04	1.68 ± 0.07	2.01 ± 0.03	2.21 ± 0.05	1.81 ± 0.04	1.75 ± 0.02	1.61 ± 0.04	1.58 ± 0.02	1.52 ± 0.03	1.48 ± 0.02	1.63 ± 0.02	1.54 ± 0.01
Derivatives of caffeic acid-6	0.97 ± 0.01 ^#^	0.86 ± 0.01	nd	0.59 ± 0.03	0.43 ± 0.01	nd	0.76 ± 0.01	0.86 ± 0.01	0.70 ± 0.01	0.92 ± 0.01	nd	0.58 ± 0.02	0.43 ± 0.01
Derivatives of caffeic acid-7	0.47 ± 0.01 ^#^	0.41 ± 0.01	nd	nd	0.39 ± 0.01	nd	0.35 ± 0.01	nd	0.37 ± 0.01	0.48 ± 0.01	nd	nd	0.51 ± 0.01
Catechin	6.25 ± 0.17 **	5.73 ± 0.12	6.38 ± 0.07	6.02 ± 0.07	5.87 ± 0.07	5.92 ± 0.11	6.01 ± 0.09	6.24 ± 0.17	6.52 ± 0.16	6.08 ± 0.12	5.92 ± 0.31	5.86 ± 0.63	6.17 ± 0.27
Methoxy apigenin	3.95 ± 0.07 ^#^	3.48 ± 0.09	3.35 ± 0.07	4.01 ± 0.07	3.75 ± 0.12	4.06 ± 0.17	3.86 ± 0.13	3.39 ± 0.09	3.52 ± 0.08	3.68 ± 0.07	4.12 ± 0.24	3.86 ± 0.41	3.73 ± 0.21
The amount of flavonoids	13.50	12.56	12.76	13.34	13.11	12.94	13.13	13.07	13.60	13.18	13.30	12.79	1298
The amount of hydroxycinnamic acids	109.11	94.48	102.22	108.72	101.73	99.95	108.48	108.98	109.91	108.16	104.61	106.29	97.85
The amount of identified substances	164.26	147.91	152.91	162.16	152.53	155.27	162.92	162.92	165.67	160.51	158.29	159.37	150.62

According to the *t*-test, the differences in content of biologically active substances (BAS) in extracts with and without amino acids were considered to be ^#^ extremely statistically significant (*p* < 0.0001), * not statistically significant (*p* = 0.34–0.97), ** very statistically significant (*p* = 0.0004–0.015), or *** not quite statistically significant (*p* = 0.08); nd—not detected.

**Table 2:** The quantitative content of the main groups of BAS in the modified extracts of motherwort.

Extract	The Quantitative Content, mg/100 g, $\bar{x}\pm \Delta \bar{x}$		
	Polyphenols Calculated with Reference to Pyrogallol	Flavonoids Calculated with Reference to Hyperoside	Iridoids Calculated with Reference to Harpagide Acetate
With the motherwort tincture	167 ± 11 *	17 ± 4 **	57 ± 2 ***
With lysine	163 ± 6	16 ± 1	51 ± 3
With aspartic acid	162 ± 8	19 ± 2	58 ± 2
With leucine	166 ± 7	15 ± 4	52 ± 1
With alanine	169 ± 10	16 ± 3	53 ± 2
With phenylalanine	174 ± 5	14 ± 4	55 ± 1
With glycine	161 ± 13	12 ± 3	61 ± 3
With valine	168 ± 5	15 ± 4	56 ± 2
With histidine	158 ± 9	16 ± 2	57 ± 2
With glutamic acid	164 ± 11	18 ± 1	54 ± 1
With arginine	161 ± 8	13 ± 2	60 ± 2
With cysteine	169 ± 7	15 ± 3	59 ± 2
With methionine	165 ± 4	16 ± 4	55 ± 1

According to the *t*-test, the differences in content of BAS in extracts with and without amino acids were considered to be not statistically significant at * *p* = 0.64, ** *p* = 0.27, and *** *p* = 0.18.

**Table 3:** Characteristics of total ultraviolet (UV) absorption spectra of extracts of motherwort.

Extract	λ_1 max_	A_1_	λ_2 max_	A_2_	A_1_/A_2_
With the motherwort tincture	285	0.158	326	0.161	0.98
With arginine	281	0.537	324	0.517	1.04
With valine	282	0.273	326	0.267	1.02
With phenylalanine	280	0.428	331	0.360	1.19
With alanine	281	0.362	324	0.345	1.05
With aspartic acid	292	0.211	327	0.214	0.99
With glycine	288	0.509	328	0.509	1.00
With lysine	288	0.582	326	0.576	1.01
With leucine	289	0.467	328	0.473	0.99
With histidine	278	0.541	327	0.542	1.00
With cysteine	285	0.458	326	0.434	1.06

**Table 4:** The results of the study of the effect of motherwort extracts on the behavioral responses of laboratory rats in the open-field test.

The Experimental Group of Animals	The Number of the Actions Recorded							
	Crossings	Upright Postures	Peeking into Holes	Exploratory Activity: ∑ Postures + Holes	Defecation	Urination	Washing	Emotional Response: ∑ Boluses + Urination + Grooming
Control	28.67 ± 1.75	5.67 ± 0.52	3.83 ± 0.82	4.75 ± 1.14	2.50 ± 0.84	2.00 ± 0.63	1.83 ± 0.75	2.11 ± 0.76
Extract								
With lysine	39.17 ± 4.45 *	8.00 ± 0.63 *^,##^	4.00 ± 0.89	6.00 ± 2.22	1.17 ± 0.41 *^,#^	1.67 ± 0.52	1.17 ± 0.41	1.33 ± 0.49 *^,#^
With aspartic acid	38.67 ± 14.53	8.0 ± 1.79 **	2.83 ± 0.75	5.42 ± 3.06	1.17 ± 0.41 *^,#^	1.50 ± 0.55	1.33 ± 0.82	1.33 ± 0.59 *^,#^
With leucine	17.00 ± 6.20 *^,#^	3.00 ± 1.67 *^,#^	2.17 ± 0.75 *	2.58 ± 1.31 *^,#^	1.17 ± 0.41 *^,#^	1.17 ± 0.41 **^,##^	1.33 ± 0.52	1.22 ± 0.43 *^,#^
With alanine	18.17 ± 1.17 *^,#^	4.17 ± 0.75 *^,##^	1.67 ± 0.52 *^,##^	2.92 ± 1.44 *^,##^	3.83 ± 1.17 **^,##^	1.17 ± 0.41 **^,##^	0.83 ± 0.41 **^,##^	1.94 ± 1.55
With phenylalanine	33.33 ± 4.97	7.83 ± 4.88	6.33 ± 0.82 *^,#^	7.08 ± 3.42 **	2.50 ± 0.84	1.33 ± 0.52	1.50 ± 0.84	1.78 ± 0.88
Control	21.67 ± 1.21	4.67 ± 0.82	2.83 ± 0.75	3.75 ± 1.22	2.33 ± 0.82	2.00 ± 0.63	1.67 ± 0.82	2.00 ± 0.77
Extract								
With glycine	62.50 ± 1.87 *^,#^	7.83 ± 1.17 *	5.17 ± 1.17 *^,##^	6.50 ± 1.78 *^,##^	1.33 ± 0.52 **^,#^	1.00 ± 0.00 **^,##^	1.17 ± 0.41	1.17 ± 0.38 *^,#^
With valine	71.33 ± 4.80 *^,#^	7.17 ± 1.17 *	5.00 ± 0.63 *^,##^	6.08 ± 1.44 *	1.50 ± 0.55 ^#^	0.83 ± 0.41 *^,#^	1.17 ± 0.41	1.17 ± 0.51 *^,#^
With histidine	48.50 ± 2.74 *^,#^	6.33 ± 1.03 **	2.50 ± 0.55	4.42 ± 2.15	1.67 ± 0.52^#^	1.33 ± 0.52	1.33 ± 0.52	1.44 ± 0.51 **^,#^
With glutamic acid	46.67 ± 2.16 *^,#^	5.50 ± 1.05	1.50 ± 0.55 *	3.50 ± 2.24	1.00 ± 0.00 **^,#^	1.00 ± 0.00 *^,#^	1.33 ± 0.52	1.11 ± 0.32 *^,#^
Control	23.50 ± 1.87	4.50 ± 0.84	2.50 ± 1.05	3.50 ± 1.38	2.83 ± 0.75	2.17 ± 0.75	2.17 ± 0.75	2.39 ± 0.78
Extract								
With arginine	42.50 ± 2.88 *	6.67 ± 1.21 *	5.83 ± 1.47 *^,#^	6.25 ± 1.36 *^,##^	2.33 ± 1.03	1.17 ± 0.41 **^,##^	1.67 ± 0.82	1.72 ± 0.89 **
With cysteine	39.00 ± 7.95 *	6.33 ± 1.03 *	4.00 ± 1.10 **	5.17 ± 1.59 **	1.67 ± 0.52 **^,##^	1.50 ± 0.55	1.33 ± 0.52	1.50 ± 0.51 *^,#^
With methionine	29.67 ± 3.01 *^,#^	6.83 ± 0.75 *	4.17 ± 1.17 **	5.50 ± 1.68 *	3.00 ± 0.89	1.33 ± 0.52	1.17 ± 0.41 **	1.83 ± 1.04
With the motherwort tincture (reference)	39.67 ± 1.63 *	6.17 ± 1.47 **	2.17 ± 0.75	4.75 ± 1.96	2.33 ± 0.52	2.00 ± 0.63	1.83 ± 0.75	2.06 ± 0.64

*—significant compared to the control group at *p* < 0.01; **—significant compared to the control group at *p* < 0.05; ^#^—significant compared to the reference drug group at *p* < 0.01; ^##^—significant compared to the reference drug group at *p* < 0.05.

**Table 5:** The results of the study of modified dry extracts of motherwort in the elevated plus maze test.

The Experimental Group of Animals	The Time Spent in the Closed Arm, s	The Time Spent in the Open Arm, s	The Number of Entries to the Closed Arm	The Number of Entries to the Open Arm	The Time Spent in the Center, s
Control	153.7 ± 3.2	19.8 ± 1.3	5.0 ± 0.6	4.8 ± 0.4	6.5 ± 3.0
Extract					
With lysine	66.3 ± 1.4 *^,##^	96.2 ± 4.2 *^,#^	4.5 ± 0.5	6.3 ± 0.5 ^#^	17.5 ± 3.1 *
With glutamic acid	64.5 ± 2.0 *	103.2 ± 2.5 *^,#^	6.8 ± 0.8 *^,##^	4.5 ± 0.9 **^,##^	12.3 ± 1.9 *
With phenylalanine	129.7 ± 9.6 *	32.0 ± 3.9 *^,#^	5.0 ± 0.9	6.0 ± 0.4 **^,#^	18.3 ± 7.8 **
With arginine	53.8 ± 2.5 *^,#^	105.5 ± 2.4 *^,#^	5.2 ± 0.8	5.0 ± 0.0 ^#^	20.7 ± 3.1 *^,##^
With glycine	49.5 ± 4.0 *	127.0 ± 6.7 *^,#^	4.3 ± 1.0	7.3 ± 1.0 *^,##^	5.2 ± 2.1
With alanine	144.5 ± 2.9 *	29.5 ± 3.2 *^,#^	7.3 ± 1.4 *^,##^	6.3 ± 1.4 **^,##^	6.0 ± 2.4
Control	152.5 ± 3.7	19.0 ± 2.1	4.5 ± 1.0	4.3 ± 0.8	8.5 ± 2.7
Extract					
With methionine	100.5 ± 2.3 *	67.2 ± 2.9 *^,#^	4.8 ± 1.5	5.5 ± 1.0	12.3 ± 2.4 **
With methionine	107.8 ± 5.1 *	63.3 ± 4.1 *^,#^	7.0 ± 1.4 *	5.3 ± 0.8	8.8 ± 3.6
With leucine	148.7 ± 2.4 ^##^	27.2 ± 1.9 *^,#^	6.8 ± 1.6 **	6.2 ± 0.8 *	4.2 ± 2.2 **^,##^
With histidine	51.8 ± 2.3 *^,#^	97.7 ± 3.6 *^,#^	5.7 ± 1.2	7.0 ± 1.4 *	30.5 ± 4.0 *^,#^
With valine	41.8 ± 3.9 *^,#^	114.3 ± 4.7 *^,#^	3.3 ± 1.0 ^#^	5.3 ± 1.0	23.8 ± 2.3 *^,#^
With aspartic acid	101.0 ± 2.6 *^,##^	61.0 ± 1.4 *^,#^	7.2 ± 0.8 *^,##^	4.8 ± 0.8 ^##^	18.0 ± 1.3 *^,##^
Motherwort tincture	83.7 ± 7.9 *	85.3 ± 6.7 *	5.5 ± 1.0	6.2 ± 0.8 *	11.0 ± 6.2

*—*p* ≤ 0.01 in relation to the indicator in the group of control animals; **—*p* ≤ 0.05 in relation to the indicator in the group of control animals; ^#^—*p* ≤ 0.01 in relation to the indicator in the group of animals taking the motherwort tincture; ^##^—*p* ≤ 0.05 in relation to the indicator in the group of animals taking the motherwort tincture.

## Data Availability

The study did not report any data.
